# Sources of variability in the measurement of *Ascaris lumbricoides* infection intensity by Kato-Katz and qPCR

**DOI:** 10.1186/s13071-017-2164-y

**Published:** 2017-05-25

**Authors:** Alice V. Easton, Rita G. Oliveira, Martin Walker, Elise M. O’Connell, Sammy M. Njenga, Charles S. Mwandawiro, Joanne P. Webster, Thomas B. Nutman, Roy M. Anderson

**Affiliations:** 1 Helminth Immunology Section, Laboratory of Parasitic Diseases, National Institute of Allergy and Infectious Disease, National Institutes of Health, Bethesda, MD 20814 USA; 2 Department of Infectious Disease Epidemiology and London Centre for Neglected Tropical Disease Research (LCNTDR), Faculty of Medicine, Imperial College London St Mary’s Campus, London, W2 1PG UK; 3 The Eastern and Southern Africa Centre of International Parasite Control (ESACIPAC), Kenya Medical Research Institute, Nairobi, Kenya; 4 Department of Pathobiology and Population Science and London Centre for Neglected Tropical Disease Research (LCNTDR), The Royal Veterinary College, Hawkshead Lane, Hatfield, Hertfordshire AL97TA UK

**Keywords:** Kato-Katz, qPCR, *Ascaris lumbricoides*, Diagnostics, Deworming, Impact evaluation, Soil-transmitted helminths, Measurement error

## Abstract

**Background:**

Understanding and quantifying the sources and implications of error in the measurement of helminth egg intensity using Kato-Katz (KK) and the newly emerging “gold standard” quantitative polymerase chain reaction (qPCR) technique is necessary for the appropriate design of epidemiological studies, including impact assessments for deworming programs.

**Methods:**

Repeated measurements of *Ascaris lumbricoides* infection intensity were made from samples collected in western Kenya using the qPCR and KK techniques. These data were combined with data on post-treatment worm expulsions. Random effects regression models were used to quantify the variability associated with different technical and biological factors for qPCR and KK diagnosis. The relative precision of these methods was compared, as was the precision of multiple qPCR replicates.

**Results:**

For both KK and qPCR, intensity measurements were largely determined by the identity of the stool donor. Stool donor explained 92.4% of variability in qPCR measurements and 54.5% of observed measurement variance for KK. An additional 39.1% of variance in KK measurements was attributable to having expelled adult *A. lumbricoides* worms following anthelmintic treatment. For qPCR, the remaining 7.6% of variability was explained by the efficiency of the DNA extraction (2.4%), plate-to-plate variability (0.2%) and other residual factors (5%). Differences in replicate measurements by qPCR were comparatively small. In addition to KK variability based on stool donor infection levels, the slide reader was highly statistically significant, although it only explained 1.4% of the total variation. In a comparison of qPCR and KK variance to mean ratios under ideal conditions, the coefficient of variation was on average 3.6 times larger for KK highlighting increased precision of qPCR.

**Conclusions:**

Person-to-person differences explain the majority of variability in egg intensity measurements by qPCR and KK, with very little additional variability explained by the technical factors associated with the practical implementation of these techniques. qPCR provides approximately 3.6 times more precision in estimating *A. lumbricoides* egg intensity than KK, and could potentially be made more cost-effective by testing each sample only once without diminishing the power of a study to assess population-level intensity and prevalence.

**Electronic supplementary material:**

The online version of this article (doi:10.1186/s13071-017-2164-y) contains supplementary material, which is available to authorized users.

## Background

As attention shifts from morbidity control for soil-transmitted helminths (STHs) to transmission interruption, accurate and precise measures of both prevalence and intensity of infection when both are low is of high importance [1]. Assessing the beneficial impact of interventions is complicated by the absence of reliable, inexpensive, and sensitive diagnostics to track changes in the prevalence and intensity of helminth infections after multiple rounds of treatment [2, 3]. The Kato-Katz (KK) smear microscopy method is commonly used in resource-limited settings because it is simple, quantitative, and can detect *Schistosoma mansoni*, liver flukes and STHs [4–6]. The current paper compares the sources of variability in traditional KK microscopy with the newer and more sensitive qPCR diagnostic method [7–9].

Studies on variability in measurement (measurement error) can be used to assess the value of additional sampling effort. Several recent studies have examined the benefit of additional sampling effort in increasing KK sensitivity for STHs and schistosomes [10–12]. A study of KK for the diagnosis of *S. mansoni* in a highly endemic area of Côte d’Ivoire found that intra-specimen variation was higher than day-to-day variation in egg counts, though day-to-day variation became more important after treatment when infections were light. This study concluded that taking repeated measurements from a single stool was an acceptable way of measuring infection intensity in high transmission areas [13]. A recent review discusses the sources of variability in egg excretion and egg counting procedures, addressing KK as well as other techniques [14].

Since statistical power depends on effect size, it will always require less sampling effort to detect large changes compared to small ones (in drug efficacy or in infection intensity or prevalence, for example). More precision is required to reliably detect small changes. This can be achieved by increased sampling effort or by using more precise diagnostic techniques. Whether additional sampling effort is worth the additional cost will depend on the measure of interest. For example, a recent meta-analysis found that minimal sampling effort was sufficient to reliably estimate infection intensity, but that the accuracy of prevalence estimates significantly increased with additional effort [15].

Both biological and technical factors reduce the accuracy and precision of faecal egg counts, as measured by the standard KK, as a proxy for an individual’s underlying worm burden. Biological factors include person-to-person differences in EPG (eggs per gram of stool) resulting from, for example, differences in stool volume and consistency, and thus not necessarily reflecting true differences in helminth infection levels. Stool volume and consistency can vary by day, season and region, and by a person’s age and diet [16, 17]. The host immune system may also influence the longevity of worms, and their egg output [18, 19]. Furthermore, infection with male worms and pre-patent female worms cannot be assessed by diagnostics based on egg counts, including both KK and qPCR.

Technical errors in EPG measurement result from factors such as slide quality, egg clumping in stool and human error [20–22]. Egg counts are especially imprecise in particularly dry or wet (diarrheic) stools; for *S. mansoni*, dry stools may produce egg counts up to seven times greater than wet stools from the same person [23] (because KK is based on a specific volume that fits inside a standardized template rather than on a specific mass). Clumping of eggs in stool can add to variability in measurements, and homogenization of faecal samples is recommended for detection of *S. mansoni* eggs, though evidence of clumping has not been conclusively demonstrated for *Ascaris lumbricoides*, *Trichuris trichiura* or hookworm eggs [21, 22]. Finally, rapid and accurate assessments of egg counts, and species identification, require training and experience and are naturally subject to human error [7, 20].

The variability of qPCR results has also been examined in a range of contexts (see Table 1). Some of the sources of variability in qPCR are similar to those that affect KK. Since qPCR is largely a measure of STH egg DNA in stool [24], qPCR will likely fail to detect the presence of a male or a pre-patent female worm. It is not known whether qPCR regularly detects material from adult worms, as discussed in a recent study of qPCR for schistosomes [25]. qPCR has additional unique sources of variability, which do not affect KK; the efficiency of DNA extraction [26, 27], imperfect pipetting [28], and the DNA target amplified [24]. These technical sources of variability are controlled in two key ways during the qPCR process. The constant concentration of a passive reference dye in each well provides an independent reference against which the cycle threshold (Ct) is calculated, and “standard curves” (a set of five samples of known helminth DNA concentration) are used to standardize the helminth DNA quantities calculated from measured Cts. As with EPG measurements by KK, variability influences the smallest detectable difference between samples. Vaerman and colleagues found that a two-fold DNA concentration difference was the smallest observable difference [29], while another study estimated that a 1.3 to 3.2-fold difference could be detected [30].

**Table 1:** Sources of variation in Kato-Katz (KK) and qPCR measurement of helminth eggs in stool

	Kato-Katz	qPCR
Biological	Worm burden of host and per-worm egg output [49, 53, 54]; Stool volume and consistency [55, 56]; Egg clumping [21, 22, 57]	Same as shown at left for KK
Technical	Slide readability, technician’s skill [20, 56]; Degradation of eggs on slide over time (particularly important for hookworms) [22, 58, 59]	Efficiency of DNA extraction [26, 27]; Pipetting error [28]; Target and primer DNA sequences [24]; Reaction conditions

This study investigated the sources and implications of variability in the measurement of *A. lumbricoides* infection intensity by KK and qPCR. We sought to attribute variability in infection intensity measurements to specific biological and technical factors. Implications for monitoring and evaluation studies are discussed.

## Methods

### Stool and worm collection

The data collection in Kenya and processing has been described in detail previously [9]. Egg count data were based on slides read as part of an epidemiological survey of individuals in five villages in Bungoma County, western Kenya, at two time-points, 3 months apart. During this survey, two slides were made from each stool collected, and each slide was read once (each slide by a different technician). An additional 200 mg of each stool was cryopreserved for qPCR. A subset of this dataset from the baseline survey, for which full metadata on explanatory variables was available, was used in the regression analysis described below. This subset of the baseline survey data is described in more detail in Table 2.

**Table 2:** Sample sets used for the examination of Kato-Katz and qPCR technical variability

Sample	Questions addressed
34 Kato-Katz slides containing *Ascaris lumbricoides* eggs, each read by five technicians. These 34 slides are a subset of 50 slides read by five technicians (other than the 34, these slides did not contain *A. lumbricoides* eggs).	To assess reader-to-reader differences in measurement.
16 of the 34 slides have corresponding qPCR results (note: only readings by the first four readers are included in this sample, to make it comparable to qPCR samples read in quadruplicate).	To compare variability in measurements from the same sample between qPCR and Kato-Katz.
351 slides from 158 individuals at baseline, each read by one of nine readers. Two thirds were from the first stool collected from each individual, and the remainder were from the second stool. Twenty percent (72/351) of these slides were judged to be poorly made, based on being poorly spread or totally opaque. Twenty-three percent (82/351) of slides were from people who later expelled a worm following treatment.	To assess the relative contribution of different factors (listed in Additional file 1: Table S1) to the variability in egg intensity measurements by Kato-Katz.
Four qPCR quantity measurements from each of 284 samples that were tested by qPCR and had at least one positive reading for *A. lumbricoides*.	Additional information about deviation of individual measurements from the mean measurement, to supplement the data from the four stool samples discussed directly below.
Four de-identified stool samples, split into 11 pieces each. Each stool sample was extracted after being split into 11 different pieces, and each of these 11 samples was run in four wells on each of three plates. These four people are not associated with a number of worms expelled.	To assess the relative contribution of different factors (listed in Additional file 1: Table S1) to the variability in egg intensity measurements by qPCR.
383 *A. lumbricoides* worms expelled at baseline and 141 expelled at follow-up.	Examine the reliability of worm expulsion by calculating its sensitivity, and explore whether the sizes of worms expelled at follow-up suggest that egg excretion might have been suppressed following baseline treatment.

An additional dataset was created from the independent readings made by five different technicians of 34 slides that contained *A. lumbricoides* eggs. Of these 34 egg-positive slides read by multiple technicians, 16 were prepared from 10 stool samples that were also analysed by qPCR. This dataset is described further in Table 2.

After the baseline survey, all individuals in the study villages were offered treatment with 400 mg albendazole (ALB). The first wave of treatment included all individuals who were egg-positive for *A. lumbricoides*. At the time of the first wave of treatment, Community Health Workers (CHWs) collected the entire stool produced by each participant in this subsample, providing new plastic collection containers every 24 hours for 7 days. This length of time was chosen based on the results of a pilot study (and on previous studies [31–33]), which indicated that approximately 80% of the total number of worms in each person would be expelled during this time.

Visible *A. lumbricoides* worms were isolated in the field laboratory, and their weight, length and sex were recorded. The determination of sex was based on morphology, where small worms with a curved tail were identified as male, as described elsewhere [34–36]. They were then stored frozen at −15 °C. At the second time-point (3 months after the first treatment), worms were collected over a 2-week period, in order to attempt to collect 100% of the worms expelled. Stool and worm samples were shipped frozen to the NIH in Bethesda, MD, USA for further analysis.

### Repeated measurement of egg intensity in stool by qPCR

DNA extraction and subsequent qPCR analysis were standardized in a number of ways: the weight of stool analysed was measured precisely, and the methods used here allow for samples to be robotically extracted and processed as a batch. DNA extraction and qPCR were performed at the NIH.

In order to examine variability due to the DNA extraction process and qPCR, stool samples (of approximately one gram each) from four individuals (de-identified and referred to as samples A through D) were each split evenly by weight into 11 Precellys Soil grinding SK38 2 ml tubes (Bertin Technologies, Montigny-le-Bretonneux, France). DNA was then extracted as previously described [9]. As part of this extraction and qPCR methodology, 2 μl of a stock solution containing an internal amplification control (IAC) plasmid [37] was added to each replicate during the extraction process. When the IAC did not amplify during qPCR, this was an indication that the detection of DNA was inhibited, and thus false negative results might have occurred when the same sample was tested for STH DNA. However, if the bead-beating was insufficient to free STH DNA from hard egg shells, or the small amount of STH material in the sample was below the limit of detection, a false negative result for that STH could still occur, even if the IAC DNA amplified in that sample.

Extracted DNA was eluted in 200 μl of sterile water in order to provide sufficient material for repeated testing. Reactions took place in 10 μl volumes (including 2 μl DNA template) with both master mix and template being pipetted by a Beckman Coulter Biomek NXP robotic liquid handler (Beckman Coulter, Brea, CA) into 384-well plates. DNA from each extraction was added to four wells. Primer and probe sequences have been previously described [38]. Each plate was run on the Viia7™ Real-Time PCR System under standard fast chemistry settings previously described [8]. Thus, each sample was tested a total of 132 times (11 replicates extracted, each run in four wells on three different plates). An additional plate was run to test for the IAC plasmid, as failure to detect the plasmid (or detection at an abnormal Ct) could signal failure of the DNA extraction to efficiently remove substances that could inhibit the qPCR.

DNA was extracted from the head of a single adult *A. lumbricoides* worm and quantified using a NanoDrop (Thermo Scientific, Wilmington, DE, USA). This suspension of *A. lumbricoides* DNA was serially diluted tenfold, to make up five dilutions covering a range of DNA concentrations. Each of these five standards was run in quadruplicate on each plate. Cycle thresholds (Cts, the number of cycles after which the level of detection of the target sequence exceeds background noise) for each sample were converted into DNA quantities based on standard curves. Earlier detection results from a higher concentration of helminth DNA; thus low Cts correspond with high helminth DNA concentrations.

### Statistical analysis

Statistical analysis was performed using Prism version 6.0 (GraphPad, La Jolla, CA), R version 3.2.1 (R Foundation for Statistical Computing, Vienna, Austria, 2015), Microsoft Excel for Mac 2011 (Microscoft Corporation, Redmond, WA) and JMP 12 (SAS, Cary, NC). Means are arithmetic unless otherwise specified.

Random-effects regression models were developed and run in R using the *lme4* package and the function glmer (which fits generalized linear mixed-effects models). Because egg counts are overdispersed integers (variance greater than the mean), a random effects term was included for each individual observation, permitting extra-Poisson variation among counts measured from the same individual [39–41]. This random effects term was not included in the model for qPCR, as that dataset was the combination of four approximately normally distributed sets of measurements from four different individuals.

The regression model for qPCR included as random effects: the identity of the stool donor, the extraction, on which plate and in which well the sample was run, and if the Internal Amplification Control (IAC) was detected in the normal range. The regression model for KK egg counts included as random effects: the identity of the stool donor, whether adult worms were ever collected from the donor, whether the stool was from the first or second sample collected from the donor, which parasitologist read the slide, whether the slide was sufficiently well-spread and transparent to be read easily, and whether a long time elapsed between slide preparation and reading. These factors are outlined and described further in Additional file 1: Table S1.

The Akaike Information Criterion (AIC) was used to assess the parsimony and adequacy of the complete model (using the full list of explanatory variables measured) versus partial models made by removing one explanatory variable at a time (to identify the ‘best’ model). Partial and full models were also compared using a likelihood ratio test to calculate the Chi-square *P*-value between the two models.

In order to further investigate the added precision gained from repeated qPCR measurement of each sample in multiple wells, each raw measurement from four de-identified stool samples A-D was compared to the mean of the four measurements made from the same DNA solution from the same extraction. Percent differences from the mean were calculated for each raw measurement, except for those where any one of the four measurements failed to detect any DNA (because the data are discontinuous around zero).

To look at the precision gained by having repeated readings of individual KK slides, the same analysis was done for the 34 slides read by multiple readers. Only readings by the first four readers were used, in order to mirror the four technical replicates available for the qPCR data described in the previous paragraph. The percent difference between each raw egg count and the average of four egg count readings was mapped against the average egg count.

To further examine the precision gained from additional sampling effort in KK, variability from reader-to-reader, day-to-day and slide-to-slide was also compared. The analysis of reader-to-reader differences took into account readings of the 34 slides by all five technicians. Because the data for the regressions was limited to samples that had complete metadata, the slide-to-slide and day-to-day sample sizes are larger, allowing for a more complete analysis of these variables. The slide-to-slide dataset contains 2715 comparisons of two slides from the same stool, and the day-to-day datasets (for both KK and qPCR) contain 216 comparisons of two average measurements from two different days. Slide-to-slide and day-to-day correlations were estimated from Spearman’s rank correlation coefficients in Prism. For reader-to-reader comparisons, a Friedman test (a non-parametric alternative to a repeated measures ANOVA) was run in Prism. Reader-to-reader differences were analysed using the dataset of 34 *A. lumbricoides* egg-positive slides because that many independent readings by different readers were not available in the main survey dataset used for the regressions.

For the 16 egg-positive slides (out of 34) read by multiple readers, for which there was a qPCR result from the same stool, the mean and variance of egg counts was calculated based on readings by four independent technicians. The mean and variance of the qPCR measurements was calculated based on the results from the four wells tested for each sample. The coefficient of variation (CoV) by both methods, and the ratio of the CoV for KK measurements to the CoV for qPCR measurements, was calculated for each stool. Since these intensive repeated measurements were made on the same stools using the contrasting KK versus qPCR techniques, this analysis enabled the comparison of precision in methods.

## Results

### Variability of qPCR measurements

**Fig. 1:**
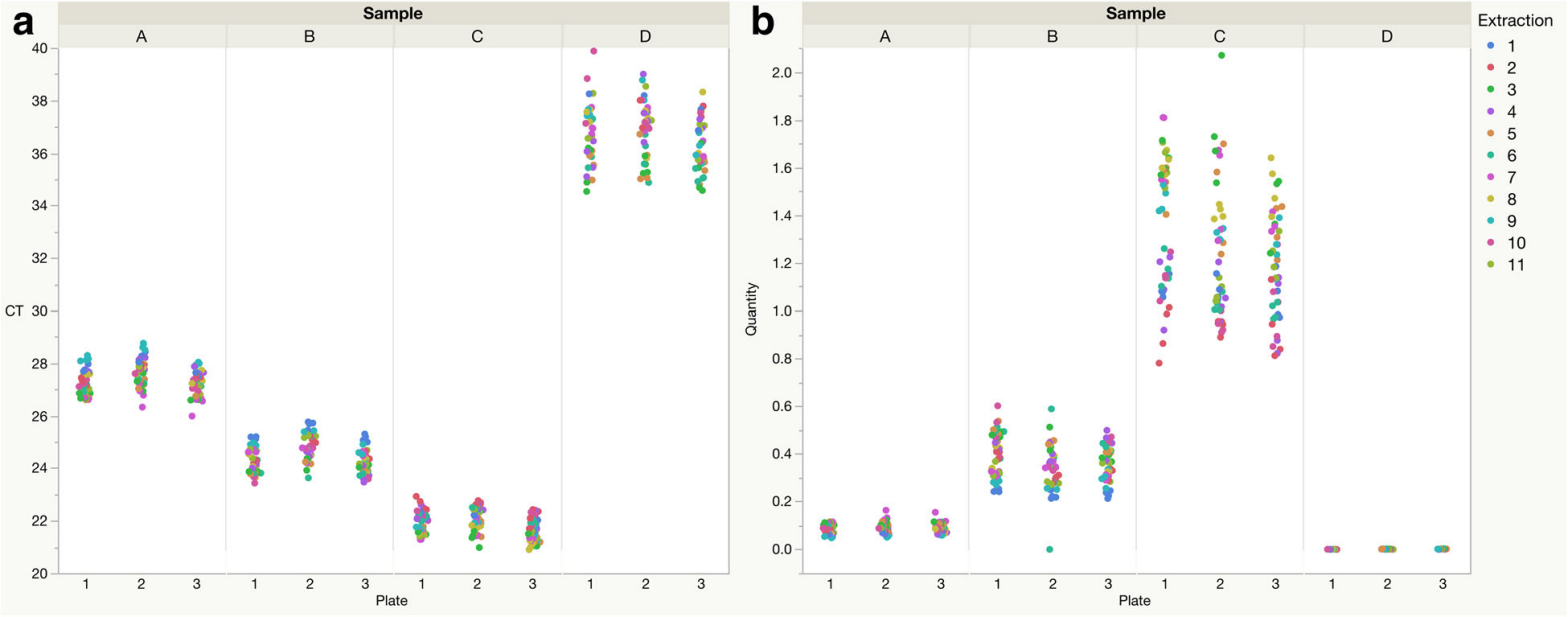
Repeated extractions and qPCR plate runs enable observation of measurement variability. Four samples (A-D) of approximately 1 g were each split evenly by weight between 11 tubes. DNA was extracted from each of these 44 replicate samples using a robotic protocol. Each replicate sample was run in quadruplicate, on each of three plates. **a** Cts are shown on the Y-axis. The difference between the highest and the lowest of the Cts for these samples is 2.8, 2.3, 2.0 and 5.3, respectively. **b** Each Ct from (**a**) was converted into a DNA concentration (in ng/μl) using the standard curve on that plate. The ranges of DNA concentrations for these four samples were 0.11, 0.60, 1.3 and 0.0028, respectively

Repeated testing of four samples (A-D) for *A. lumbricoides* DNA was used to isolate the contribution of biological and technical factors to measurement variability (Fig. 1). Each of the 11 extractions from each stool was tested in quadruplicate on each of three qPCR plates, for a total of 132 tests per stool sample. The range of outcomes covered 2–3 Cts for the samples with average Cts in the range of 21–28 (samples A-C), as shown in Fig. 1a. For sample D, which had a higher average Ct (37), the measurements of these replicates covered a range of five Cts (Fig. 1a). When these Cts were converted into DNA quantities, measured in ng/μl using the standard curves, the three samples with higher infection intensities had ranges covering approximately the same magnitude as the average value (Fig. 1b). For the sample with the higher Cts, the results cover a range more than twice the magnitude of the average value. The average *R* ^2^ linear correlation coefficient for the Cts of the standard curves versus the log_10_ DNA quantity was 97%. Though not perfect, this indicates that Ct can be used to accurately predict DNA quantity.

To examine the contribution of the factors shown in Fig. 1, a regression was performed with stool donor, extraction, plate and “well” as explanatory variables (see Additional file 1: Table S1). The stool donor contributed the most information, with 92.4% of the variance being explained by this variable. These differences likely represent true differences in infection level between different individuals. The extraction was the next most important factor, explaining 1.7% of the total variance (Table 3). The level of internal amplification control (IAC) detected contributed an additional 0.7%. IAC measures the efficiency of the extraction, so these two extraction-related variables combined explained 2.4% of the total variance. The regression model was worse (significant Chi-square *P*-value and higher AIC value, shown in Table 3) when the qPCR plate variable was omitted, but plate explained only 0.2% of the total variance, meaning that its impact, though significant, is not necessarily important. Since there was no significant improvement in the regression model fit when the “well” variable was omitted (Chi-square *P*-value was not significant and the AIC value was lower than for full model), “well” itself was not an important contributing factor to the measurement of *A. lumbricoides* DNA by qPCR. Since there was no measurement of the number of worms infecting each of the four stool donors, it was not possible to include worm number in the regression model. Any variability that could be explained by each donor’s worm count is likely included in the variability attributed by the model to differences between stools from different individuals.

**Table 3:** Variance components show relative importance of factors for repeated measurements by qPCR

	% variance explained	AIC when item excluded, relative to 13,947 for full model	Chi-square value (1 degree of freedom) and *P*-value for comparison with full model
Stool donor	92.4	15,322	*χ* ^2^ = 1376, *P* < 0.0001****
Extraction	1.7	14,037	*χ* ^2^ = 92, *P* < 0.0001****
Plate	0.2	13,955	*χ* ^2^ = 10, *P* = 0.001***
Well	0.0	13,945	*χ* ^2^ = 0, *P* = 1
IAC level	0.7	13,949	*χ* ^2^ = 4, *P* = 0.04 *
Residual	5.0		

*Abbreviation*: *AIC* Akaike information criterion

*P ≤ 0.05; **P ≤ 0.01; ***P ≤ 0.001; ****P ≤ 0.0001

**Fig. 2:**
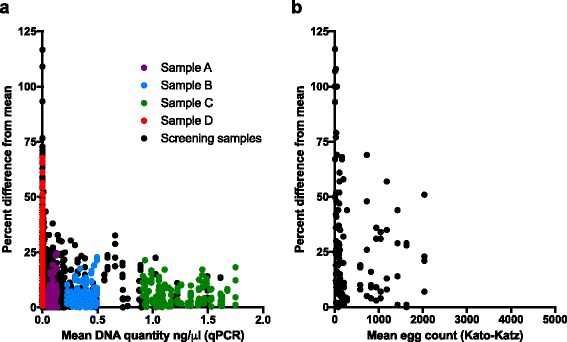
Percent difference between raw qPCR and Kato-Katz measurements and average of four technical replicates, versus the average measurement. **a** For samples A-D, the percent difference between each measurement relative to the average of four technical replicates (wells on the same qPCR plate from the same extraction) was calculated. This was plotted against the average of the four measurements. The percent difference from the mean of each of four readings was also plotted against the mean for 284 stool samples tested by qPCR during the screening phase where at least one reading was positive for *A. lumbricoides* (shown in *black*). **b** The percent difference of each raw read from the mean of four reads from each slide was plotted against the mean of the four egg counts. Though each of these slides was read by five readers, only four were analyzed here, in order to be as comparable as possible to the four technical replicates analyzed in (**a**). The scale of the X-axis was chosen to represent a similar range of egg intensities as seen in (**a**)

Since “well” was not an important factor in the regression model, it follows that testing each sample in multiple wells should not provide a significant increase in precision. For samples A-D, each of 33 measurements was made in quadruplicate (replicated in four wells). When we calculated the difference between each raw measurement and the average of all four measurements, for samples A-C, 95% of all measurements fell within 15% of the mean measurement (Fig. 2a). However, for sample D, the individual with the lightest *A. lumbricoides* infection, deviance from the mean measurement was much greater. This suggests that, below 0.01 ng/μl, qPCR intensities are not as reliable as they are above 0.05 ng/μl, at which point well-to-well differences are stable. Though additional infections are detected with each additional well, since the qPCR methodology only counts a sample as positive if ¾ wells are positive (to reduce false positives), additional testing is not likely to change the measured prevalence either.

### Variability in KK measurements

**Fig. 3:**
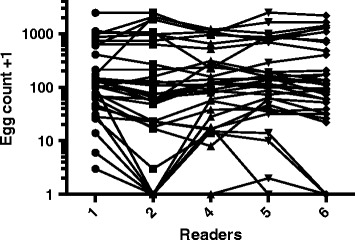
Between-reader differences evident in controlled experiment. Fifty slides were selected from the pool of slides being assessed during one of the screening phases, and recoded to make them anonymous. Five readers (numbered on the X-axis) read each of these slides independently. Of the 50 slides, at least one reader identified an egg on 34 slides. Each set of horizontal connected dots represents readings from one of these 34 slides by different readers. The Y-axis is on a log scale to enable visualization across the range of egg counts represented here

Turning to variation due to technical errors for KK, potential differences in egg counts among readers were examined in a controlled experiment whereby each of five readers made an independent assessment of the number of eggs on each of 34 slides containing *A. lumbricoides* eggs. As seen in Fig. 3, the readings of these slides from some technicians were significantly different (Friedman statistic 13.73, *P* = 0.0082). This difference was most marked between reader #2 and readers #1 and #5.

**Fig. 4:**
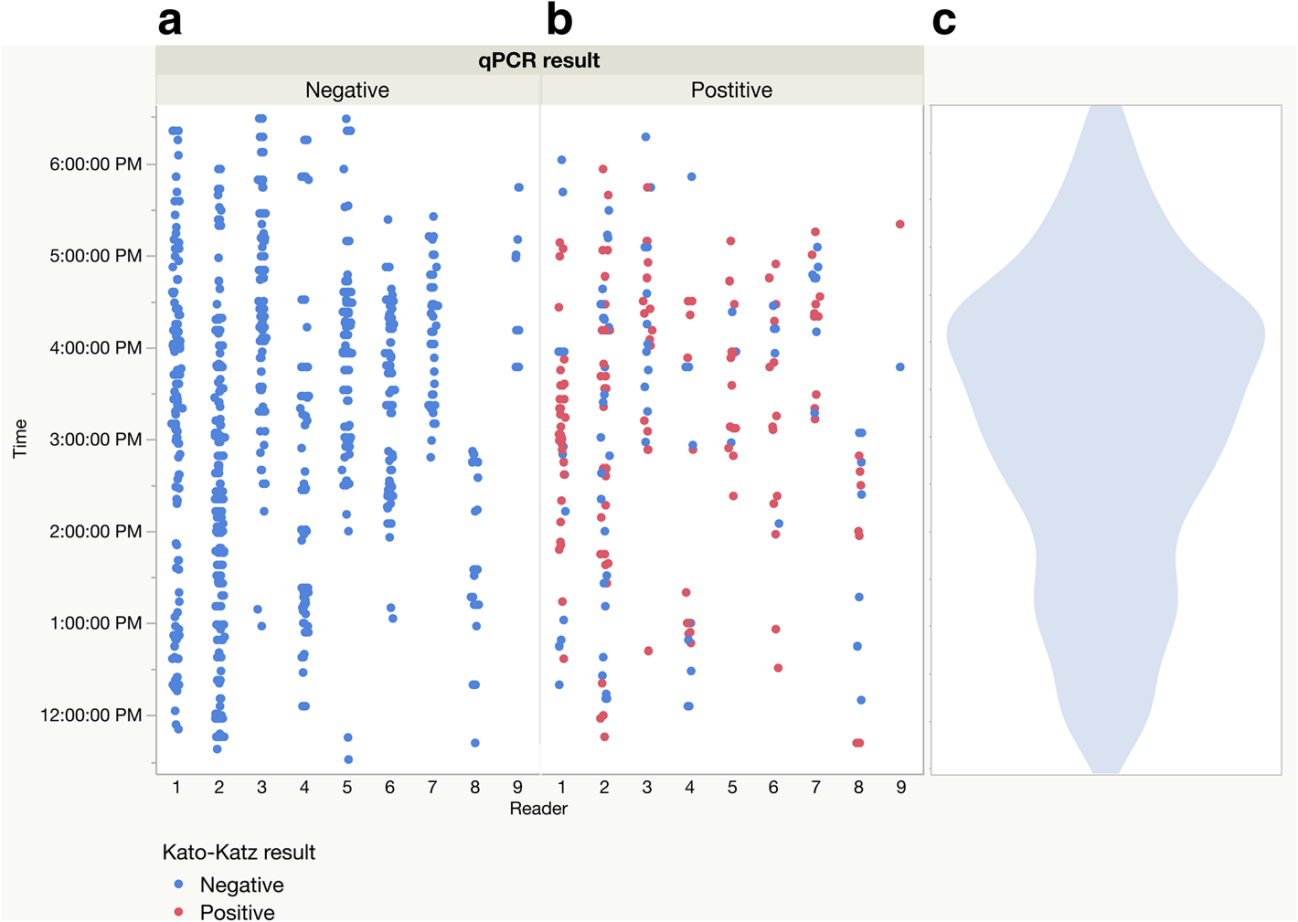
Kato-Katz ability to detect *Ascaris lumbricoides* infections shows no pattern between readers or at different times of day. Slides from the baseline time-point are spread along the Y-axis based on the time of day at which they were read. In both panels (**a**) and (**b**), samples are shown in *blue* if the sample was negative for *A. lumbricoides* by KK, and *red* if the sample was positive for *A. lumbricoides* by KK. Samples are shown above the code for the technician who read that slide. The violin plot in panel (**c**) shows that the core slide-reading period was 2 pm to 5 pm

In the field setting, other factors in addition to the slide reader come into play. We sought to examine the relative importance of various factors in terms of their contribution to the measured egg count. To illustrate, *A. lumbricoides* egg counts recorded were stratified in Fig. 4 by the qPCR result for the same slide, which technician read the slide, and the time at which it was read. Slides were read between 11:30 am and 6:30 pm. Time could be an important variable for two reasons: technicians might be fatigued at the end of the day, and the samples read at the end of the day are likely to have been processed outside the intended window of time after they were prepared. All of the samples later found to be negative for *A. lumbricoides* by qPCR (shown on the left panel of Fig. 4) were negative by KK as well. As can be seen by the density of points, some readers worked constantly throughout the day, while others spent the morning and early afternoon on slide preparation, and only began reading slides later in the afternoon. In the middle panel of Fig. 4, it can be seen that some qPCR-positive slides were read as KK positive and negative for *A. lumbricoides* throughout the day by all readers. This could be because eggs were missed, were not visible, or because the section of stool on that slide did not contain an egg.

In order to examine the relative contributions of different factors, a random-effects regression model was fitted to the data with KK egg count as the outcome variable. Explanatory variables were: the stool donor, whether the donor ever expelled *A. lumbricoides* worms, the day the donor provided the stool, the slide reader, the time between slide preparation and reading, and slide quality (whether or not the slide was sufficiently transparent and evenly spread to allow for easy visualization of helminth eggs).

As shown in Table 4, the percentage of variance attributable to the stool donor is larger than that attributable to any other variable. More than half of the total variance (54.5%) was attributable to the individual who donated the stool. Whether or not the individual who donated the stool ever expelled *A. lumbricoides* worms explained an additional 39.1% of variation in egg counts (Table 4). This is encouraging given that egg counts are widely used in epidemiological studies of STHs as a surrogate of the worm burden in an individual. None of the following variables explained any variation in egg counts: how well the slide was made; how much time passed between when the slide was made and when it was read; and/or which day the slide was from.

**Table 4:** Sources of variability in repeated KK screening measurements for *Ascaris lumbricoides*. All variables are described in detail in Additional file 1: Table S1

	% variance explained	AIC when item excluded, relative to 1742	Chi-square value (all with 1 degree of freedom) and *P*-value for comparison to full model
Stool donor	54.5	1883	*χ* ^2^ = 143, *P* < 0.0001****
Worms expelled from donor	39.1	1738	*χ* ^2^ = 0, *P* = 1
Slide reader	1.4	1756	*χ* ^2^ = 16, *P* < 0.0001****
Day	0	1740	*χ* ^2^ = 0, *P* = 1
Slide quality	0	1740	*χ* ^2^ = 0, *P* = 1
Time	0	1740	*χ* ^2^ = 0, *P* = 1
Sample ID	4.9		

*Abbreviation*: *AIC* Akaike information criterion

*P ≤ 0.05; **P ≤ 0.01; ***P ≤ 0.001; ****P ≤ 0.0001

As confirmation of which factors were important, the AIC values are listed for the model minus each factor individually. The AIC values are relatively constant, but go up (showing the model performing worse) when stool donor is omitted. Sample ID is not omitted, because it is essential to modelling the overdispersed distribution of repeated egg counts measured from the same individual.

### Worm expulsion

qPCR results add additional information, especially about low-intensity infection, that was not available when only KK was used to test for *A. lumbricoides* infection. However, only the observation of *A. lumbricoides* adult worms can provide direct information about an individual’s worm burden. We have shown previously that qPCR and egg counts are equally good predictors of the number of worms expelled [9].

However, worm counts also provide substantial information about the inaccuracies of KK and qPCR (such as by showing that worms were likely to have been growing in a person at a time when no STH eggs or egg DNA was detected). The comparison of egg and worm counts also provides substantial information about how unreliable worm counts are: such as how insensitive worm expulsion (using benzimidazoles) is for the diagnosis of *A. lumbricoides*.

A total of 383 *A. lumbricoides* worms were collected from 85 individuals at baseline, and 142 *A. lumbricoides* worms (from 25 individuals) were collected at follow-up, 3 months after the first study treatment. Among people who expelled worms at baseline, 10% were egg-negative by KK, and 5% were qPCR-negative. Expelled worms were only found in 56% of individuals who were egg-positive for *A. lumbricoides* by KK (results were similar for those positive by qPCR). The average raw egg count (which could be multiplied by 24 to obtain the EPG) was higher in the egg-positive individuals from whom worms were collected (411 eggs) compared to egg-positive individuals from whom no worms were ever collected (59 eggs).

Worm collection was discontinued after 7 days at baseline, but at follow-up, stools continued to be collected until 14 days after treatment. At follow-up, the last worm was observed on the 11th day after treatment (Additional file 2: Figure S1). Expulsion timelines at baseline were similar across age ranges, but at follow-up, worms from individuals ages 6–9 appeared to be expelled earlier than those from people of older and younger ages.

At baseline, there was no observable trend in sex ratio, worm weight or worm length by day of expulsion. However, at follow-up, it became clear that female worms were expelled towards the beginning, and male worms continued to be expelled into the second week (Additional file 2: Figure S1). This resulted in worm weight and length decreasing with time, as the sex ratio shifted towards greater representation of the smaller male worms.

Worm sexing was performed in the field and confirmed in the laboratory for a subset of the worms collected. After accounting for miss-categorizations, 72% of worms were estimated to be female. The fact that morphological identification of the sex of *A. lumbricoides* worms is difficult means that an accurate assessment of the number of eggs expelled per female worm is difficult to calculate without transporting worms to the laboratory for sex determination by dissection. Unfortunately, only some of the technicians working on this study recognized and recorded the presence of unfertilized eggs, so records of unfertilized eggs are not analysed here.

**Fig. 5:**
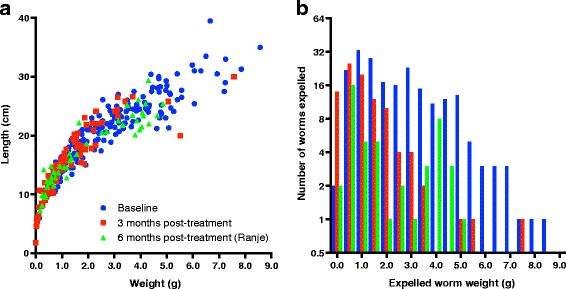
Worm dimensions show population of heavy worms was reduced at follow-up. Each worm’s length and weight was measured on the day it was collected. There was a longer gap in time between worms collected in Ranje (the pilot study village), as worms there were collected during the pilot, and then only after the follow-up data collection had finished in the four main study villages. **a** The length of each worm is plotted against the width of that worm, to show the concentration of small worms at all time-points and the small number of heavy, long worms still present at the post-treatment time-points. **b** The weights of these same worms are plotted as interleaved histograms, to show that the distribution of worms shifts to the left post-treatment

Worm length plateaued at about 30–35 cm, but worms near this maximum length weighed anywhere from 5 g to nearly 9 g. At the 3-month follow-up time-point, there were fewer worms longer than 5 cm (red and green points compared with blue points in Fig. 5a). However, there were three worms (red points) in this very large category. Since it takes 2 to 3 months after eggs are ingested for female worms to begin producing eggs, it is likely that these three worms, as well as many of the other large worms collected at follow-up, were present at the baseline time-point as well. As shown in Fig. 5b, the distribution of worm weights shifted left between the baseline time-point (blue) and the follow-up time-points. The three largest worms from the 3-month time-point can be seen in red in this figure.

### Comparison in variability by method at comparable egg intensities

Of the 34 slides read by multiple technicians (shown in Fig. 3), 16 were prepared from 10 stool samples that were also analysed using qPCR. Mean, variance and coefficient of variation (CoV) measurements for these samples are shown in Table 5. These variances represent variability due only to reader for KK and only to pipetting or qPCR machine error for qPCR. The average of all the ratios of CoVs was 3.6, meaning that the CoV was approximately 3.6 times larger for these samples by KK than by qPCR. Thus, across infection intensities, we estimated that the variance as measured by KK was 3.6 times larger, relative to the mean, than the variance by qPCR (relative to the mean). However, the true variance in KK and qPCR measurements will also depend on the quality of the KK and qPCR methodology, and on the intensity of STH infection in a study area. If qPCR methodology is not standardized at a sufficient level, it may not be comparable to the results obtained in this lab at the NIH.

**Table 5:** Mean and variance calculated from four technical replicate measurements from each of 16 slides

Sample number	qPCR		KK		qPCR	KK	CoV KK/CoV qPCR^a^
	Mean	Variance	Mean	Variance	Coefficient of variation		
1	0.1300	0.00035684	375	10,061	0.15	0.27	2
2	0.0004	0.00000003	8	78	0.46	1.18	3
3	0.0285	0.00000085	116	425	0.03	0.18	5
4	0.0285	0.00000085	117	140	0.03	0.10	3
5	0.0019	0.00000005	3	5	0.12	0.68	6
6	0.0067	0.00000060	76	418	0.12	0.27	2
7	0.1300	0.00035684	313	13,847	0.15	0.38	3
8	0.0149	0.00000174	140	44	0.09	0.05	1
9	0.0011	0.00000004	12	108	0.17	0.88	5
10	0.0149	0.00000174	210	341	0.09	0.09	1
11	0.0606	0.00019056	94	582	0.23	0.26	1
12	0.0634	0.00019056	104	246	0.22	0.15	1
13	0.0634	0.00000766	95	475	0.04	0.23	5
14	0.0634	0.00000766	114	2025	0.04	0.39	9
15	0.0835	0.00002502	123	1294	0.06	0.29	5
16	0.0835	0.00002502	97	1348	0.06	0.38	6

Since some of the KK slides used in this analysis were prepared from the same stool (slides A and B), but each stool was only analyzed once by qPCR, some mean and variance qPCR measurements are shown in duplicate in this chart. The subset of samples used for this analysis is described in Table 2

^a^ Coefficient of variation (variance divided by mean) of Kato-Katz measurements divided by the coefficient of variation of measurements from the same stool

Variability in intensity measurement can be visualized as percent differences from the mean of four repeated measurements, shown in Fig. 2. The X-axes represent similar egg-intensity ranges, though the 34 slides read by multiple technicians do not cover the full range observed in this setting. This figure shows that qPCR and KK precision are similar for egg counts near zero, but qPCR measurements quickly stabilize as egg intensities increase, so that most qPCR measurements fall within 20% of the mean of four measurements (Fig. 2).

### Biological variability in egg counts from multiple stool samples from the same donor

**Fig. 6:**
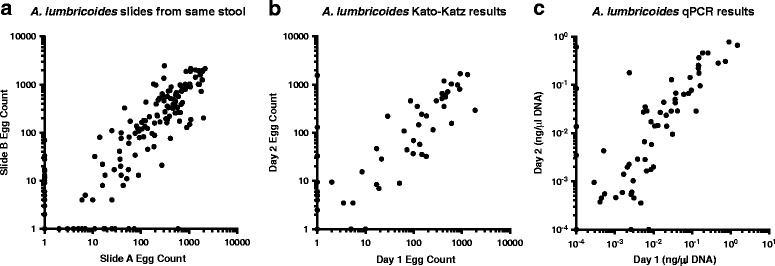
Slide-to-slide and day-to-day variation evident but limited. **a** Two slides (“A” and “B”) were made from each of 2715 baseline stools collected and read by different technicians. **b**, **c** 216 people had stool samples collected on multiple days and analysed by both KK and qPCR. The measurements from the second day are plotted against the measurements from the first day in each graph

During data collection in field settings, it is common practice to make two slides from each stool, and to have them read by different readers [42]. The Spearman correlation for slides A and B from each of the 2715 stool samples examined here is 0.84 (Fig. 6a). Though there is a strong correlation between these different readings from the same stool, there is still substantial variation between the slides, due to either the measurement process or to the difference in the number of eggs in different pieces of the same stool.

Measurements of egg output are likely to change even more from day-to-day than from slide-to-slide. Day-to-day variation in *A. lumbricoides* intensity was reflected by egg output, as measured by either qPCR or KK (Fig. 6b, c). The Spearman correlation coefficient, r, for *A. lumbricoides* measurements by KK (Fig. 6b) was 0.87, and by qPCR (Fig. 6c) was 0.93, demonstrating a high degree of correlation among repeated measures.

## Discussion

This study sought to apportion error in the measurement of *A. lumbricoides* egg intensity to different possible sources of error. To do so, qPCR and KK results were examined under controlled conditions. While some of the variables examined contributed significantly to variability in measurements (extraction for qPCR and reader for KK in particular), the vast majority of variability depended only on which study participant donated the stool examined. This likely represents true differences in infection intensity between people. There were no worm expulsion results for comparison with the four samples tested by qPCR, where person-to-person differences explained 92.4% of variability (Table 3). Since the objective of most field studies on deworming programs is to look at variation in worm burden across people in a population, it is encouraging to find that qPCR-based measurements of individual infection intensities are not masked by technical sources of variation. For KK, person-to-person differences explained 54.5% of variability, and whether or not each person had ever expelled a worm explained an additional 39.1% of variability, for a combined total of 93.6%. Hence, compared with qPCR, a similar proportion of variability in intensity measurements by KK is explained by individual differences in infection, rather than technical variables such as reader or slide quality (Table 4).

This does not necessarily contradict previous findings that differences between laboratories can be important [20, 43]. Instead, it may mean that when there are so many different sources of variation in a field-based dataset such as this one, it is very difficult to pinpoint specific sources of error. There may be additional technical issues (not measured here) that could explain additional technical variability.

This does not mean, however, that KK and qPCR are able to identify *A. lumbricoides* egg intensities with a very high level of precision. The range of Ct values was relatively tight for lower Ct values, representing higher *A. lumbricoides* DNA concentrations (Fig. 1a). However, when these values were converted into DNA concentrations, the exponential transformation means that there was a wider range of estimates for the samples with higher helminth DNA concentrations (Fig. 1a, b). For the four samples analysed, the size of the range was approximately equal to the mean for each sample. Thus, it appears that anything smaller than an approximately two-fold difference in helminth DNA concentration cannot be interpreted as a meaningful difference in concentration. This is similar to the conclusion derived by others that a two-fold change is the smallest change detectable by qPCR [29].

Understanding the level of measurement variance (error) can help determine how many samples, or repeat testing of samples, to collect or perform in order to get a specified level of precision [28]. Since each raw intensity measurement by qPCR is within approximately 20% of the mean of four measurements from the same sample, except at very low infection intensities, intensity measurements are reasonably reliable at most infection intensities observed (Fig. 2). This means that the cost of qPCR testing could be reduced by testing each sample only once, allowing for more samples to be tested on a given plate. As KK tests cost approximately US$2.00 per child, it may be difficult to scale up use of a molecular test if the cost per individual tested is substantially higher than this figure [1]. Even if the higher cost of qPCR slows investment in its use, it may be the case that using qPCR or another diagnostic with high sensitivity could save governments money in the long term, as a result of aiding them in making cost-effective policy decisions [44].

Researchers have previously used measures of variability to compare diagnostics for helminth infection intensity, such as FLOTAC, KK and McMaster [45–47]. These studies generally found that FLOTAC was more precise than the other methods, usually by comparing the coefficient of variation. Our study found that, for ten stool samples repeatedly tested by both methods, reader-to-reader differences for a single slide resulted in an average of 3.6× higher coefficients of variation than well-to-well differences obtained by qPCR measurements (Table 5). Since the standard equation for sample size is proportional to sample variance [48], this could mean that 3.6 times more samples would be needed for a study using KK than for the same study if qPCR were used. However, this ratio will depend on the rigor of both the KK and the qPCR protocols used in other studies.

Many of the biological factors that cause KK measurements to have a high variance in repeat measurements from the same individual have been examined extensively in previous studies [13, 17, 49, 50]. Whether measurement variability was studied within stool samples, between stools taken from the same individual on different days or from stools from different individuals, the negative binomial distribution described well each source of variation [49]. However, there was still a strong non-parametric correlation between different slides from the same stool (Fig. 6a), and different stools from the same individual (Fig. 6a, b). This suggests that (at least for relative quantifications) day-to-day and slide-to-slide variation may not have been a major problem in the data collection for this study.

Whether an individual expelled worms was a large predictor of egg intensity (though the model was not significantly worsened by its removal, as seen in Table 4, as the variability described by this variable is likely wholly included in the stool donor variable). However, other person-to-person differences between stool donors were even more important explanatory variables (Table 4). Some of these person-to-person differences, though not due to measurement error, could be a result of biological sources of error, such as the impact of stool consistency on EPG. It is also possible that the worm burden measured in this expulsion study was so prone to error itself that it is a flawed measure of an individual’s worm burden, especially because the long expulsion timeline likely reduced compliance with stool collection.

## Conclusions

qPCR was previously found to be much more sensitive for the detection of low intensity infections in the dataset used here, and equally as predictive of the number of *A. lumbricoides* worms expelled as KK [9]. Here, we show that little of the variability causing overdispersion in intensity measurements by both diagnostic tools can be attributed to specific known sources. Instead, the vast majority of differences in intensity measurements can be attributed to real biological differences in intensity among people. Since the majority of variability in qPCR measurement is due to the stool donor, and only a small additional part is due to technical factors, when resources are constrained, it is not necessary to run qPCR samples in more than one well each. More research would be useful to confirm this result due to its potential importance for deworming program evaluations. It may be surprising that sampling on multiple days was not found to be critically important for KK in this study, though other studies on the benefit of repeated sampling by KK of individuals have also found that in many circumstances, collecting multiple stool samples from individuals is not necessary to get an accurate and sensitive KK result [51, 52]. Though the costs of consumables could be reduced by testing each sample only once by qPCR, setting up laboratories in endemic areas where qPCR is not yet available will still be slowed by the required initial investment in equipment, and training in equipment maintenance and use. This work has focused primarily on *A. lumbricoides*, as stools with *A. lumbricoides* eggs were readily available. However, because KK is less sensitive for hookworm than for *A. lumbricoides*, there might also be greater differences in precision between qPCR and KK for the measurement of hookworm egg intensities than we found for *A. lumbricoides*. Thus, we postulate that qPCR could be even more useful for the detection and quantification of infection with hookworm than with *A. lumbricoides*. Measuring changes in helminth egg intensity is necessary for evaluating the impact of a mass deworming program. Though several studies have recently compared the sensitivity of different qPCR protocols with KK and other microscopic techniques, we hope this study will provide useful information on precision for future impact evaluation studies. Both diagnostic tools appear able to provide useful and technically consistent intensity measurements, though the inherent variability of each technique must be accounted for in sample size calculations. Since qPCR, as used here, appears to be 3.6 times as precise as KK (and ~1.4 times more sensitive [9]), and remains similarly precise even when no replicates are run, this technique will likely provide better information about *A. lumbricoides* infection, especially in low-prevalence settings.

## Additional files

Additional file 1: Table S1. Categorical variables included in regressions (DOCX 101 kb).

Additional file 2: Figure S1. *Ascaris lumbricoides* expelled each day after treatment, by sex. Worms were collected between the 2nd and 11th days post-treatment. The total number of male and female worms (assessed in the field by morphology) expelled is shown for each day. Female worms appear to peak on day four, whereas male worms were expelled continuously throughout the expulsion period (PDF 42 kb).

## Abbreviations

AIC: Akaike information criterion
EPG: Eggs per gram of stool
IAC: Internal amplification control
KK: Kato-Katz microscopic technique
MDA: Mass drug administration
qPCR: Quantitative real-time polymerase chain reaction
STH: Soil-transmitted helminth
