# Supplementary material for: Sources of variability in the measurement of Ascaris lumbricoides infection intensity by Kato-Katz and qPCR
Source: Parasit Vectors. 2017 May 25;10:256. doi: 10.1186/s13071-017-2164-y (PMC5445470; doi:10.1186/s13071-017-2164-y)
Supplement: Supplementary file 1 — Categorical variables included in regressions (DOCX 101 kb). [file 13071_2017_2164_MOESM1_ESM.docx]

**Additional file 1: Table S1.** Categorical variables included in regressions

|  | Variable | Explanation |
| --- | --- | --- |
| KK | Stool donor | Accounting for differences in infection intensity between individuals. This biological variable represents real differences between people, not measurement error. |
|  | Worms | Whether a person ever expelled a worm, confirming their infection. |
|  | Day | Whether the stool is from the first day the individual was tested or the second day. |
|  | Slide reader | The identity of the parasitologist who read the slide. |
|  | Slide quality | The slide was categorized as difficult to read if it was not transparent to light or was not spread into an even disc. Ratings used here were made by a single person. |
|  | Time | Slides that were in the top 25% in terms of the time between when the sample was made and when it was read were categorized as having taken a long time to be read. |
| qPCR | Stool donor | See above |
|  | Extraction | Each stool was split into 11 pieces, and extracted separately. This variable captures both clumping of eggs in stool and the extraction process. |
|  | Well | Each extraction of each stool is added to four wells (referred to as testing these samples in quadruplicate). This variable captures variability resulting from pipetting and from the qPCR machine. |
|  | Plate | Samples were run on 384-well plates, so only this many wells can be tested at a time. |
|  | Internal amplification control level | Two microliters of plasmid solution is added to each sample during the extraction. qPCR is later run to check the concentration of the plasmid that is detectable in the final DNA solution. Samples that had an IAC Ct reading in the highest quintile of all readings were considered to be an indication that inhibition of the qPCR might have been occurring. |
