# Supplementary figures and images for: Sources of variability in the measurement of Ascaris lumbricoides infection intensity by Kato-Katz and qPCR
Source: Parasit Vectors. 2017 May 25;10:256. doi: 10.1186/s13071-017-2164-y (PMC5445470; doi:10.1186/s13071-017-2164-y)

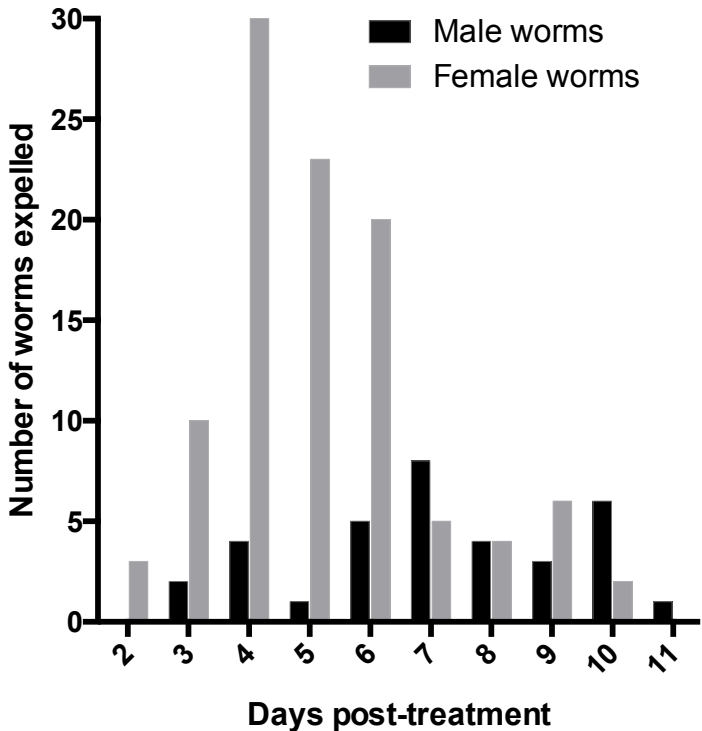

Supplement: Supplementary file 2 — Ascaris lumbricoides expelled each day after treatment, by sex. Worms were collected between the 2nd and 11th days post-treatment. The total number of male and female worms (assessed in the field by morphology) expelled is shown for each day. Female worms appear to peak on day four, whereas male worms were expelled continuously throughout the expulsion period (PDF 42 kb). [file 13071_2017_2164_MOESM2_ESM.pdf]
